# Surgery for Killian-Jamieson diverticulum: a report of two cases

**DOI:** 10.1186/s40792-020-0789-0

**Published:** 2020-01-13

**Authors:** Kohei Saisho, Satoru Matono, Toshiaki Tanaka, Naoki Mori, Haruhiro Hino, Masahiro Fujisaki, Masashi Nakagawa, Fumihiko Fujita, Yoshito Akagi

**Affiliations:** Department of Surgery, Kurume University School of Medicine, 67, Asahimachi, Kurume City, Fukuoka Prefecture 830-0011 Japan

**Keywords:** Killian-Jamieson diverticulum, Pharyngoesophageal diverticulum, Diverticulectomy, Diverticulopexy

## Abstract

**Background:**

Killian-Jamieson diverticulum (KJD) is a rare diverticulum arising from a muscular gap in the anterolateral wall of the proximal cervical esophagus. The first choice of treatment for KJD remains controversial due to its rare incidence. Here, we report two cases of KJD for which we performed different surgery: diverticulectomy in one case and diverticulopexy in the other.

**Case presentation:**

Case 1 involved a 58-year-old woman presenting progressive pharyngeal discomfort for the past year. She was diagnosed as KJD using endoscopic and radiographic findings. She underwent diverticulectomy with cricopharyngeal and proximal esophageal myotomy. Staple line leakage developed at 1 month after surgery and was successfully treated conservatively. At 5 months after surgery, she was asymptomatic. Case 2 involved a 77-year-old woman presenting dysphagia for the past 2 years. She had a history of bilateral breast cancer and had hypertension, asthma, and osteoporosis. Taking her age and medical history into account, we selected diverticulopexy with cricopharyngeal and proximal esophageal myotomy. The postoperative course was uneventful. At 2 years after surgery, she remained free of dysphagia.

**Conclusion:**

The first choice of surgery for KJD is diverticulectomy. In a high-risk patient, diverticulopexy is a reasonable treatment. We recommend the addition of myotomy as a part of any surgical treatment.

## Background

**Fig. 1:**
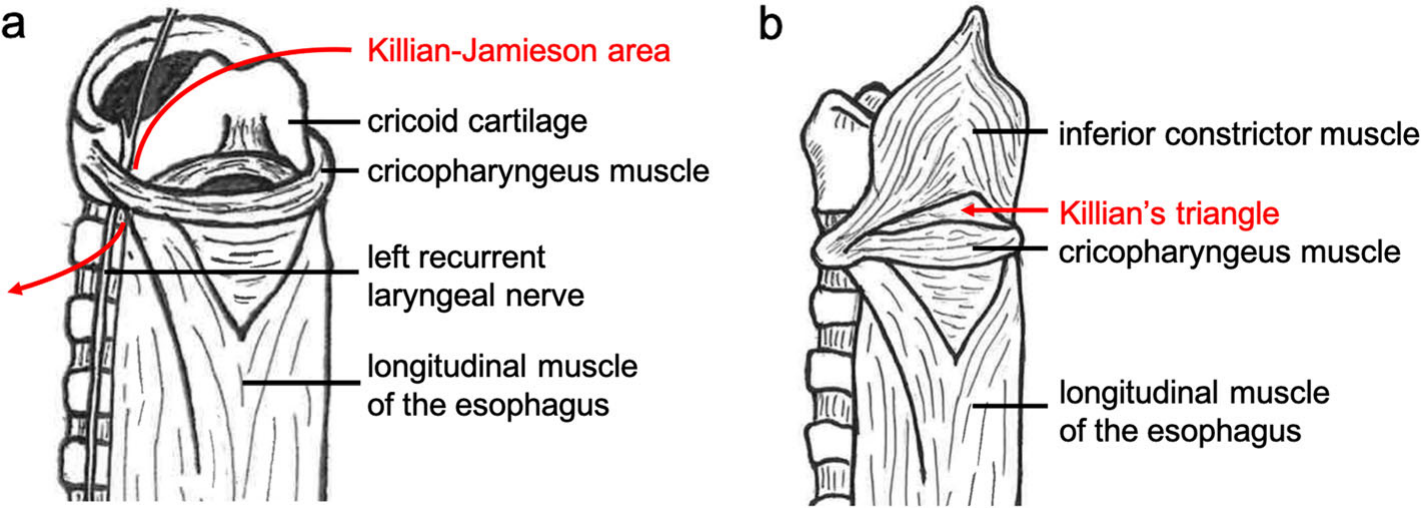
Illustration of the muscular gaps as the origin of pharyngoesophageal diverticulum. **a** Killian-Jamieson diverticulum arises from the muscular gap in the anterolateral wall of the proximal cervical esophagus just below the cricopharyngeus muscle and superolateral to the longitudinal muscle of the esophagus (Killian-Jamieson area). It is very close to the entry point of the recurrent laryngeal nerve into the larynx. **b** Zenker’s diverticulum arises from the muscular gap in the posterior aspect below the inferior pharyngeal constrictor muscle and above the cricopharyngeus muscle (Killian’s triangle)

Killian-Jamieson diverticulum (KJD) is a rare diverticulum arising from a muscular gap in the anterolateral wall of the proximal cervical esophagus just below the cricopharyngeus muscle and superolateral to the longitudinal muscle of the esophagus (Killian-Jamieson area) (Fig. 1a). KJD is distinguished from Zenker’s diverticulum (ZD), which is the most common pharyngoesophageal diverticulum, arising from the muscular gap in the posterior aspect below the inferior pharyngeal constrictor muscle and above the cricopharyngeus muscle (Killian’s triangle) (Fig. 1b). The incidence rate of KJD is one fourth that of ZD [1]. KJD is very rare and is estimated to occur in only 0.025% of the population [2]. The treatment for KJD remains controversial due to its low incidence rate. Here, we report two cases of KJD in which we performed different surgery, diverticulectomy and diverticulopexy, and discussed the surgical treatment for KJD.

## Case presentation

### Case 1

**Fig. 2:**
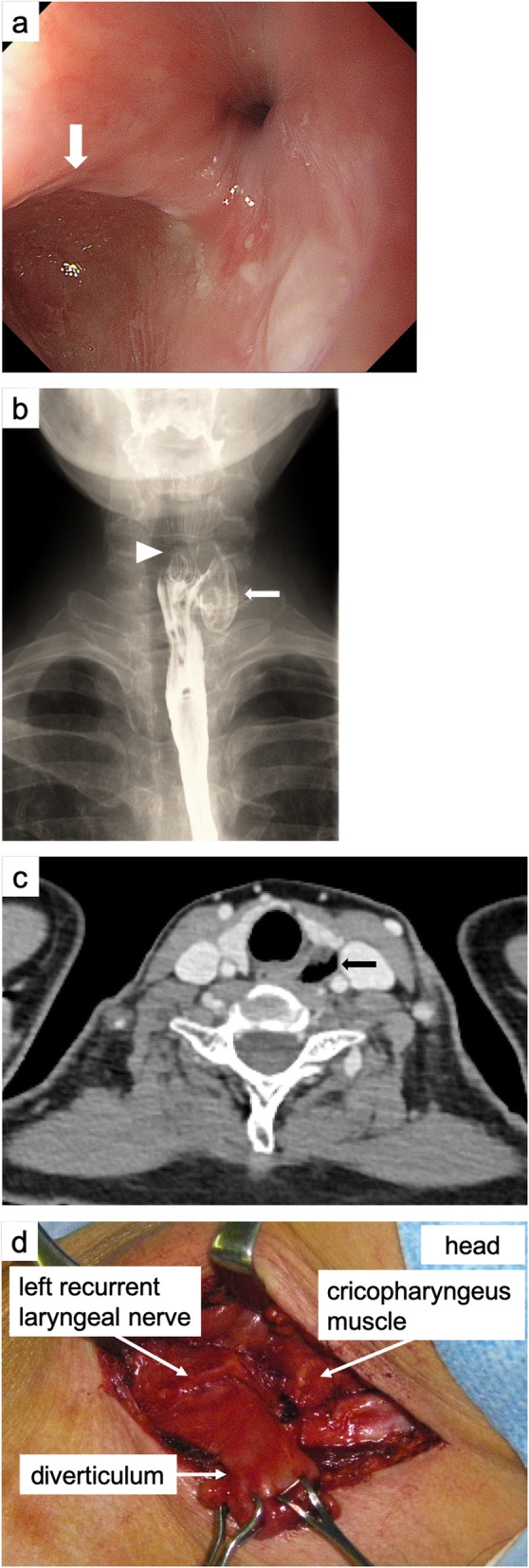
Preoperative and intraoperative findings of Case 1. **a** An esophagogastroduodenoscopy showed a diverticulum just under the esophageal orifice (white arrow). **b** An esophagogram showed a 2.5 cm-diameter diverticulum in the left side of the cervical esophagus (white arrow) and showed a cricopharyngeal bar presenting the cricopharyngeus muscle above the diverticulum (white arrowhead). **c** A cervical computed tomography showed an air-filled diverticulum arising from the left side of the cervical esophagus (black arrow). **d** Intraoperative findings. The diverticulum originated from the lateral wall of the cervical esophagus below the cricopharyngeus muscle, and the left recurrent laryngeal nerve lied very close to the base of the diverticulum.

A 58-year-old woman presented to our institution with a 1-year history of progressive pharyngeal discomfort. An esophagogastroduodenoscopy (EGD) revealed a diverticulum just under the esophageal orifice (Fig. 2a). An esophagogram confirmed a 2.5-cm-diameter diverticulum in the left side of the cervical esophagus and showed the cricopharyngeal bar presenting the cricopharyngeus muscle above the diverticulum (Fig. 2b). A cervical computed tomography (CT) showed an air-filled diverticulum arising from the left side of the cervical esophagus (Fig. 2c). The diverticulum was diagnosed as KJD based on these endoscopic and radiographic findings.

She underwent diverticulectomy under general anesthesia. She was placed in the supine position with her head rotated slightly to the right. A pad was placed underneath the shoulders to hyperextend her neck. A collar incision of left anterior neck was performed along the anterior border of the left sternocleidomastoid muscle. After incising platysma, the dissection is continued along the anterior border of the left sternocleidomastoid muscle and the carotid sheath was exposed. The sternocleidomastoid muscle and the carotid artery were retracted laterally, and the esophageal wall was exposed. The diverticulum was mobilized after identifying and preserving the left recurrent laryngeal nerve. The left recurrent laryngeal nerve was found immediately anterior to the base of the diverticulum. The diverticulum originated from the left lateral wall of the cervical esophagus below the cricopharyngeus muscle (Fig. 2d). Cricopharyngeus myotomy and 2 cm of proximal esophageal myotomy were performed. After the myotomy, the diverticulum was resected at its base using a linear stapler (ETS-Flex45, Ethicon, USA). The wound was irrigated and closed with the placement of a closed suction drain.

A gastrograffin swallow examination on postoperative day 6 revealed no evidence of leakage and the patient started oral intake. However, at 1 month after surgery, she presented with left cervical redness and pain. A gastrograffin swallow examination revealed staple line leakage. However, it was cured with fasting and percutaneous abscess drainage. At 5 months after surgery, she was asymptomatic.

### Case 2

**Fig. 3:**
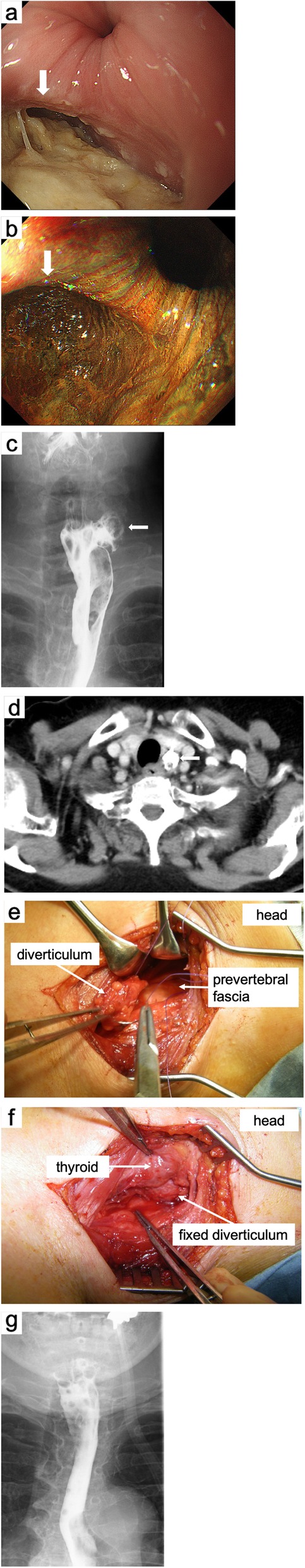
Preoperative and intraoperative findings of Case 2. **a** An esophagogastroduodenoscopy revealed a food-residue-filled pouch located at 15-16cm from the incisor (white arrow). **b** An esophagogastroduodenoscopy with iodine staining showed no cancerous lesion in the diverticulum (white arrow). **c** An esophagogram showed a 2 cm-diameter diverticulum in the left side of the cervical esophagus (white arrow). **d** A cervical computed tomography showed a diverticulum arising from the left side of the cervical esophagus. There was barium residue in the diverticulum (white arrow). **e** Procedure of fixing the diverticulum. The diverticulum was lifted and fixed to the prevertebral fascia in 5 places. 3-0 absorbable multifilament stiches were passed through the prevertebral fascia and then passed through and the diverticulum wall. **f** Appearance after diverticulopexy. **g** A gastrograffin swallow examination on postoperative day 3 revealed no evidence of leakage and the fixed diverticulum was not detected.

A 77-year-old woman presented to our institution with a 2-year history of dysphagia. She had a history of bilateral breast cancer and had hypertension, asthma and osteoporosis. An EGD revealed a food-residue-filled pouch located 15–16 cm from the incisor (Fig. 3a). Iodine staining showed no cancerous lesion in the diverticulum (Fig. 3b). An esophagogram showed a 2-cm-diameter diverticulum in the left side of the cervical esophagus (Fig. 3c). A cervical CT showed a diverticulum arising from the left side of the cervical esophagus, and barium remaining in the diverticulum (Fig. 3d). These endoscopic and radiographic findings suggested KJD.

We selected diverticulopexy, taking her age and medical history into account. The procedure of surgery was the same as Case 1 until myotomy. After the myotomy, the prevertebral fascia adjacent to pharynx was exposed, and the bottom of the diverticulum was fixed upwards to the fascia, using 3–0 absorbable multifilament stitches (Fig. 3e, f).

A gastrograffin swallow examination on postoperative day 3 revealed no evidence of leakage and the fixed diverticulum was not detected (Fig. 3g). The postoperative course was uneventful. At 2 years after surgery, she remained free of dysphagia.

## Discussion

KJD was first reported by Ekberg and Nylander in 1983 [3]. To our knowledge, in the English-language literature, there are 34 case reports (39 cases) on KJD including our present report. The 39 cases included 16 men and 23 women, with an average of 55.7 years (ranging from 2 to 88 years). The most frequent location was on the left side (72%), followed by the right side (20%) and the bilateral side (8%). Eighteen patients (46%) had dysphagia, while 12 patients (30%) were asymptomatic. In asymptomatic cases, KJD was often incidentally detected on neck ultrasonography. KJD may mimic thyroid nodule on ultrasonography, and there have been several cases in which fine-needle aspiration was performed due to misdiagnosis. Therefore, it is necessary to know the ultrasonographic feature of KJD such as the hypoechoic rim, echogenic foci and change in internal echo during swallowing [4, 5]. Nineteen reports (21 cases) mentioned the treatment for KJD (Table 1) [2, 6–22]. The most common treatment was diverticulectomy (67%), followed by endoscopic diverticulotomy (28%) and diverticulopexy (5%).

**Table 1:** Previous reports regarding the treatment for Killian-Jamieson diverticulum

Study	Age (years)	Sex	Location	Size (cm)	Treatment	Complication	Duration of follow-up (months)
Rodgers et al. [6]	53	Female	Left	N/A	Diverticulectomy + cricopharyngeal myotomy	None	24
Lee et al. [7]	55	Female	Left	1.6	Endoscopic diverticulotomy	None	3
Tang et al. [8]	51	Female	Left	1.5	Endoscopic diverticulotomy	None	2
Boisvert et al. [9]	69	Male	Bilateral	4.4 (right)/3.5 (left)	Diverticulectomy + esophageal myotomy	N/A	2
Kitazawa et al. [10]	53	Female	Left	4.5	Diverticulectomy	None	2
Chea et al. [11]	52	Female	Right	N/A	Diverticulopexy	N/A	6
Undavia et al. [12]	62	Female	Left	2.5	Diverticulectomy	None	N/A
Kim et al. [13]	68	Male	Right	10	Diverticulectomy + esophageal myotomy	None	6
Mimatsu et al. [14]	74	Female	Bilateral	1.5 (right)/4 (left)	Diverticulectomy	N/A	24
Jung and Cho [15]	54	Female	Right	N/A	Diverticulectomy + cricopharyngeal myotomy	N/A	24
Saylam et al. [16]	18	Female	Right	2	Diverticulectomy	None	12
Chang et al. [17]	46	Female	Left	5	Endoscopic diverticulotomy	None	6
	32	Male	Left	N/A	Endoscopic diverticulotomy	None	6
Bock et al. [18]	49	Male	Right	N/A	Diverticulectomy	None	12
Siow et al. [19]	49	Female	Left	N/A	Diverticulectomy	None	3
Shambayati et al. [20]	2	Female	Right	N/A	Diverticulectomy	N/A	Several months
Yun et al. [21]	88	Male	Left	5.4	Endoscopic diverticulotomy	N/A	1
Stewart et al. [2]	69	Male	Right	N/A	Diverticulectomy	None	2
Yang and Draganov [22]	71	Female	Left	2.5	Endoscopic diverticulotomy	None	2
Present study	58	Female	Left	2.5	Diverticulectomy + cricopharyngeal and esophageal myotomy	Delayed staple line leakage	5
	77	Female	Left	2	Diverticulopexy + cricopharyngeal and esophageal myotomy	None	24

*N/A* not available

Endoscopic diverticulotomy is often reported as a minimally invasive surgery for ZD [23, 24]. Endoscopic diverticulotomy is a transoral procedure that divides the septum between the esophagus and the diverticulum using a CO2 laser or stapler to drain the contents of diverticulum into the esophagus. Compared to open surgery, the advantages of this endoscopic procedure include no skin incision, shorter operative duration, minimal postoperative pain, and shorter hospitalization [23–25]. However, there are only 5 case reports on endoscopic diverticulotomy for KJD [7, 8, 17, 21, 22]. The main concern for endoscopic diverticulotomy in KJD is the risk of recurrent laryngeal nerve injury. The Killian-Jamieson area corresponds to the entry point of a recurrent laryngeal nerve into the larynx (Fig. 1a). Indeed, the recurrent laryngeal nerve lied very close to the base of the diverticulum in our present case (Fig. 2d). Endoscopic diverticulotomy does not allow identification of the recurrent laryngeal nerve, and the recurrent laryngeal nerve injury could occur transluminally during septal dissection. Therefore, in the case of KJD, open surgery which can directly confirm the recurrent laryngeal nerve is preferable. In both our present cases, recurrent laryngeal nerve palsy associated with the surgery did not occur. The indication of endoscopic diverticulotomy should be confined to the patients with contraindication for long-time general anesthesia and the patient who has a history of cervical surgery.

Open surgeries for pharyngoesophageal diverticulum include diverticulectomy and diverticulopexy. Both techniques are common for ZD. However, it is not yet clear which technique is better. We consider that the first choice of surgery for pharyngoesophageal diverticulum is diverticulectomy, regardless of the type of diverticulum. The reason is that diverticulectomy can restore the pharyngoesophageal anatomy and eliminate the possibility of carcinogenesis in the diverticulum [26]. Herbella et al. reported that the prevalence of cancer in ZD ranges from 0.3 to 7 % [27]. Although there is not yet any report of cancer in KJD, the possibility of carcinogenesis in the diverticulum should be considered when deciding on the surgical method, especially in young people. On the other hand, suture line or staple line leakage can occur in diverticulectomy, and the incidence is 1.7 to 12.7% [25, 28, 29]. The staple line leakage also occurred in one of the present cases (Case 1). The patient had no risk factor such as diabetes, chronic kidney disease, or long-term corticosteroid use. However, patients with any of these risk factors have a higher probability of leakage, and the leakage is refractory. The surgical site is close to the trachea and the carotid artery, and any prolonged inflammation may be life-threatening.

Diverticulopexy is associated with a lower risk of leakage and allows earlier peroral feeding [28–32]. Puma et al. compared the treatment outcome after diverticulectomy with those after diverticulopexy for ZD and reported that diverticulopexy achieved better symptom control and a lower morbidity rate than diverticulectomy [28]. Therefore, we consider that diverticulopexy is a reasonable treatment for high-risk patients. Although there have been only two cases of diverticulopexy for KJD including our present case (Case 2), the postoperative courses were good in both cases [11]. There is no reason why diverticulopexy is difficult to apply to KJD, and diverticulopexy for KJD may be performed as widely as for ZD. The disadvantage of diverticulopexy is that the mucosa of the diverticulum remains, which may lead to carcinogenesis [26]. In addition, Herbella et al. mentioned that diverticulopexy for a large diverticulum might be cumbersome and might create a bulky mass compressing the pharynx [32].

The necessity of cricopharyngeal and proximal esophageal myotomy for KJD is controversial. Mimatsu et al. mentioned that cricopharyngeal myotomy was not necessary in KJD because of arising below the cricopharyngeal muscle [14]. Furthermore, Siow et al. mentioned that KJD was not associated with dysfunctional sphincter muscle and did not require esophageal myotomy [19]. Nevertheless, there have been some reports that cricopharyngeal and proximal esophageal myotomy were performed [6, 9, 13, 15]. The pathogenesis of ZD is considered a morphological change in the muscles that compose the upper esophageal sphincter (UES), and hypopharynx high-pressure occurring from disorder of UES [33, 34]. Therefore, myotomy of the cricopharyngeal muscle and proximal esophageal muscle is an essential step in surgery of ZD. Stewart et al. reported a case of simultaneously occurring ZD and KJD [2], and Rubesin et al. demonstrated that 19% of KJD patients had a coexisting ZD [1]. Those findings suggest that the pathogenesis of KJD and ZD may overlap to some extent. Thus, it is considered that performing myotomy for KJD is reasonable.

## Conclusion

The first choice of surgery for KJD is diverticulectomy. However, diverticulopexy is a reasonable treatment for high-risk patients. We recommend the addition of myotomy as a part of surgical treatment. The treatment of KJD should be decided according to its anatomic feature and the patient’s individual risk factors.

## Data Availability

The authors declare that all data in this article are available within the article.

## Abbreviations

CT: Computed tomography
EGD: Esophagogastroduodenoscopy
KJD: Killian-Jamieson diverticulum
UES: Upper esophageal sphincter
ZD: Zenker’s diverticulum
